# Multiple myeloma current treatment algorithms

**DOI:** 10.1038/s41408-020-00359-2

**Published:** 2020-09-28

**Authors:** S. Vincent Rajkumar, Shaji Kumar

**Affiliations:** Division of Hematology, Mayo Clinic, Rochester, MN USA

**Keywords:** Myeloma, Targeted therapies

## Abstract

The treatment of multiple myeloma (MM) continues to evolve rapidly with arrival of multiple new drugs, and emerging data from randomized trials to guide therapy. Along the disease course, the choice of specific therapy is affected by many variables including age, performance status, comorbidities, and eligibility for stem cell transplantation. In addition, another key variable that affects treatment strategy is risk stratification of patients into standard and high-risk MM. High-risk MM is defined by the presence of t(4;14), t(14;16), t(14;20), gain 1q, del(17p), or p53 mutation. In this paper, we provide algorithms for the treatment of newly diagnosed and relapsed MM based on the best available evidence. We have relied on data from randomized controlled trials whenever possible, and when appropriate trials to guide therapy are not available, our recommendations reflect best practices based on non-randomized data, and expert opinion. Each algorithm has been designed to facilitate easy decision-making for practicing clinicians. In all patients, clinical trials should be considered first, prior to resorting to the standard of care algorithms we outline.

## Introduction

Major changes have occurred in the diagnostic criteria, staging system, response criteria, and treatment for multiple myeloma (MM) in the last decade^1^. The International Myeloma Working Group (IMWG) diagnostic criteria for MM require 10% or more clonal plasma cells in the bone marrow (and/or a biopsy proven plasmacytoma) plus any one or more myeloma defining events (MDE): end-organ damage (hypercalcemia, renal insufficiency, anemia, or bone lesions) attributable to the underlying plasma-cell disorder, bone marrow clonal plasma cells ≥60%, serum involved to uninvolved free light chain (FLC) ratio ≥100 (provided involved FLC level is ≥100 mg/L), or more than 1 focal lesion (5 mm or more in size) on magnetic resonance imaging (MRI)^2^. The current IMWG staging system for MM incorporates tumor burden and high-risk cytogenetics, and is referred to as the Revised International Staging System^3^. Updated IMWG response criteria include definitions for minimal residual disease (MRD) negativity^4^. These changes in diagnosis, staging, and response assessment have been made necessary by the rapid advances in treatment of the MM, with the arrival of several new drugs (carfilzomib, pomalidomide, daratumumab, elotuzumab, panobinostat, ixazomib, and selinexor). Numerous clinical trials provide data on best practices along the spectrum of the disease. The purpose of this current treatment algorithm is to synthesize the available data in the field and provide an evidence-based approach to the current treatment of newly diagnosed and relapsed MM.

## Classification and risk stratification

There are four major subtypes of MM that account for more than 80% of patients with the disease. They include trisomic MM, t(11;14) MM, t(4;14) MM, and MM with translocations of t(14;16) or t(14;20) referred to as MAF MM (Table 1)^5^. Secondary cytogenetic abnormalities such as deletion 17p, gain 1q, deletion 1p, deletion 13q, or monosomy 13 can occur in any of the primary cytogenetic types of myeloma, and can further modify disease course, response to therapy, and prognosis.

**Table 1:** Molecular cytogenetic classification and risk stratification of multiple myeloma (MM).

Cytogenetic abnormality	Gene/chromosome (s) affected	Risk stratification^a^
Primary cytogenetic abnormality		
Trisomic MM	Trisomies of one or more odd-numbered chromosomes	Standard risk
t (11;14) MM	CCND1	Standard risk
t (4;14) MM	FGFR3 and MMSET	High risk
MAF MM		High risk
t(14;16)	C-MAF	
t(14;20)	MAF-B	
Other		Standard risk
Secondary cytogenetic abnormality		
Gain (1q)	1q	High risk
Del (17p)	p53	High risk
p53 mutation	p53	High risk
Other		Variable

^a^Presence of any two high-risk cytogenetic abnormalities is considered double-hit MM. Presence of any three or more high-risk cytogenetic abnormalities is considered triple-hit MM.

High-risk MM is defined by the presence of t(4;14), t(14;16), t(14;20), deletion 17p, gain 1q, or p53 mutation^1^. Double-hit MM refers to the presence of any two or more high-risk abnormalities. Triple-hit MM refers to the presence of three or more high-risk abnormalities.

## Disease assessment

Bone marrow studies must be performed in all patients and must include fluorescent in situ hybridization or other more sensitive means to detect cytogenetic abnormalities. Whole-body low-dose computed tomography (CT) or positron emission tomography–CT studies are preferred over conventional skeletal surveys for bone imaging^6^. MRI scans are indicated in patients felt to have clinical smoldering multiple myeloma (SMM) to rule out focal bone marrow lesions. In patients who are in CR, MRD assessment by next-generation flow cytometry or next-generation sequencing is recommended^7,8^.

## Treatment options

Several drugs have shown activity in MM and are available for clinical use. As a result, there are numerous regimens that use two or more of these active drugs available for the treatment of MM in various settings. The major classes include alkylating agents (melphalan, cyclophosphamide) corticosteroids (dexamethasone, prednisone), immunomodulatory drugs (thalidomide, lenalidomide, pomalidomide), and proteasome inhibitors (bortezomib, carfilzomib, ixazomib). Daratumumab and isatuximab are monoclonal (M) antibodies targeting CD38, and are playing an increasingly important role in the treatment of MM. Other active approved agents include elotuzumab, a M antibody targeting the SLAMF7 antigen; panobinostat, a histone deacetylase inhibitor; and selinexor, an inhibitor of exportin-1 (XPO1). Elotuzumab, panobinostat, and selinexor do not seem to have significant single-agent activity, but appear to exert their therapeutic effect in combination with other active drugs. Anthracyclines (doxorubicin and liposomal doxorubicin) have minimal single-agent activity in MM. They are used infrequently used in the treatment of MM given availability of other active agents. However, doxorubicin is incorporated into some multi-agent combination regimens for aggressive or refractory MM.

## Treatment of newly diagnosed myeloma

**Fig. 1:**
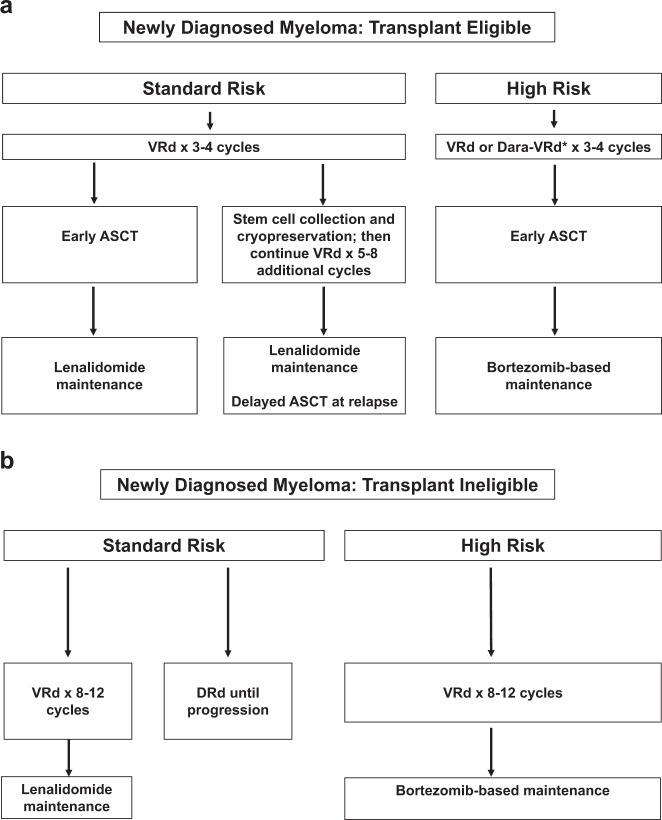
Current Treatment Algorithms for Newly Diagnosed Myeloma. Approach to the treatment of newly diagnosed myeloma in transplant-eligible (**a**) and transplant-ineligible (**b**) patients. VRd, Bortezomib, lenalidomide, dexamethasone; DRd, daratumumab, lenalidomide, dexamethasone; Dara-VRd, daratumumab, bortezomib, lenalidomide, dexamethasone; ASCT, autologous stem cell transplantation.

The two main factors that drive our approach to newly diagnosed MM are eligibility for autologous stem cell transplantation (ASCT) and risk stratification. The current algorithms for the treatment of symptomatic newly diagnosed MM based on these two factors is shown in Fig. 1. In general, eligibility for ASCT is affected by age, performance status, and comorbidities. Modern treatments can produce deep responses, and some patients can achieve MRD negative state. Although MRD negative status is associated with improved progression-free survival (PFS) and overall survival (OS), it is not the goal of therapy. There are no data from randomized trials that modifying therapy in MRD positive patients in an attempt to make them MRD negative will lead to better outcomes. Ongoing randomized trials are investigating if changing therapy based on MRD results can improve survival in MM. In the absence such data, modifying therapy based on MRD results is not recommended for clinical practice, except young patients with high-risk MM, especially double or triple-hit MM.

### Initial therapy in patients eligible for transplantation

As outlined in Fig. 1, patients who candidates for ASCT are treated with 3–4 cycles of induction therapy followed by stem cell harvest. After stem cell harvest, most patients should proceed to ASCT followed by maintenance. However in selected patients who have standard-risk MM, ASCT can be delayed until relapse. Such patients who have deferred ASCT until relapse should resume induction therapy following stem cell harvest, for a few more cycles followed by maintenance.

The preferred initial therapy for patients who are candidates for ASCT is bortezomib, lenalidomide, dexamethasone (VRd). VRd is a well-tolerated regimen with a long track record. It is associated with high overall and complete response (CR) rates. In a Southwest Oncology Group (SWOG) randomized trial, treatment with VRd led to superior PFS and OS compared with lenalidomide plus dexamethasone (Rd)^9^. A subsequent randomized trial by the Intergroupe Francophone du Myelome found that the 4-year OS rate with VRd was >80% with or without early ASCT^10^.

An important alternative to VRd in newly diagnosed MM is daratumumab, lenalidomide, and dexamethasone (DRd). DRd has shown significant efficacy in a randomized trial conducted in transplant-ineligible patients, with improved PFS compared with Rd^11^. Based on overall cost, and strength of long-term data, we prefer VRd over DRd for most patients^12^. However, DRd is a suitable alternative for patients with preexisting neuropathy or for patients who have intolerance to VRd. In high-risk patients, especially those with double-hit MM or triple-hit MM, we recommend addition of daratumumab to the standard VRd regimen (Dara-VRd). In a randomized phase II trial, Dara-VRd has shown better and deeper responses compared to VRd^13^. Another quadruplet regimen that has shown promise is daratumumab, bortezomib, thalidomide, dexamethasone (Dara-VTd). In a randomized trial, Dara-VTd was associated with improved PFS, and a trend to better OS compared to bortezomib, thalidomide, dexamethasone (VTd)^14^. Further data from phase III trials are awaited.

We do not recommend carfilzomib, lenalidomide, dexamethasone (KRd) as initial therapy. In a recent randomized trial by the Eastern Cooperative Oncology Group (ECOG), there was no significant benefit with KRd over VRd in newly diagnosed patients with standard-risk MM^15^. KRd is more expensive, and is associated with a higher risk of serious cardiac, renal, and pulmonary toxicity than VRd.

In certain settings, the treatment regimens for newly diagnosed MM have to be modified. For example, bortezomib, cyclophosphamide, dexamethasone (VCd) is our preferred regimen in patients presenting with acute renal failure due to light-chain cast nephropathy^16^. Similarly, VTd is used instead of VRd as initial therapy in countries where lenalidomide is not approved for frontline therapy^17^. In patients with primary plasma-cell leukemia or significant extramedullary disease, multi-agent combination chemotherapy such as bortezomib/dexamethasone/thalidomide/cisplatin/doxorubicin/cyclophosphamide/etoposide (VDT-PACE) may be needed initially to achieve rapid disease control^18^.

### Initial therapy in patients ineligible for transplantation

The two main options for initial therapy in patients ineligible for ASCT are VRd and DRd (Fig. 1). Melphalan-based regimens are no longer recommended due to concerns about stem cell damage, secondary myelodysplastic syndrome, and acute leukemia. VRd has shown improved OS compared with Rd, and is our preferred choice for initial therapy^9^. VRd is administered for ~8–12 cycles, followed by maintenance therapy. In frail elderly patients, a lower dose of lenalidomide and dexamethasone should be used. If therapy with VRd is not possible due to inability to travel for parenteral administration, ixazomib can be considered in place of bortezomib.

The main alternative to VRd for initial therapy in transplant-ineligible patients is DRd. DRd is approved for patients with newly diagnosed MM in the United States based on the results of a randomized trial in which PFS was found to be significantly superior to Rd^11^. MRD negative rates with DRd were also superior. The main disadvantage of DRd, is that unlike VRd where the triplet regimen is only used for a limited duration (8–12 cycles), therapy with DRd requires treatment with all three drugs until disease progression, resulting in a much more expensive and cumbersome regimen in the long term^12^. Subcutaneous availability of daratumumab may reduce the inconvenience but it still retains many of the disadvantages compared to VRd^19^.

### Dosage and supportive care considerations

There is a significant risk of peripheral neuropathy with VRd and other bortezomib-containing regimens. This can be minimized by using bortezomib in a once-weekly schedule^20,21^, and by administering the drug through subcutaneously^22^. For all regimens, dexamethasone should be used once-weekly (low-dose dexamethasone)^23^. One exception is the first 4 days of therapy in patients with acute light-chain cast nephropathy when dexamethasone can be administered daily, and then switched to once-weekly. In patients who have received prolonged lenalidomide therapy, plerixafor may be needed for adequate stem cell mobilization^24^.

All patients treated with lenalidomide need deep vein thrombosis and pulmonary embolism prophylaxis. Aspirin is adequate for most patients, but patients at higher risk of thrombosis should receive low-molecular weight heparin, warfarin, or direct thrombin inhibitors^25–27^. All patients receiving proteasome inhibitors need herpes zoster prophylaxis. We recommend prophylaxis against pneumocystis jiroveci for all patients on dexamethasone. We also recommend levofloxacin daily for the first two cycles in all patients with newly diagnosed MM^28^.

### Stem cell transplantation

#### Autologous stem cell transplantation (ASCT)

ASCT is not curative in MM, but improves median OS by ~12 months^29–36^. The treatment-related mortality rate is 1–2%. In ~50% of patients, ASCT can be done on an outpatient basis^37^. The preferred conditioning regimen for ASCT is melphalan, 200 mg/m^2^. As discussed earlier, the early use of ASCT is preferred, but in some patients with standard-risk MM, ASCT can be delayed until first relapse, primarily based on patient choice^38^. Three randomized trials conducted prior to the introduction of the VRd regimen showed that OS was similar whether ASCT is done early (after 3–4 cycles of initial therapy) or delayed (at the time of relapse as salvage therapy)^32,39,40^. A recent randomized trial using VRd as initial therapy found improved PFS but no difference in OS with early ASCT compared to delayed ASCT at the time of first relapse^10^. Thus, patient and physician preference plays an important role in deciding the timing of ASCT, especially in standard-risk patients. We still prefer early ASCT in most patients since it can be done with low risk, is logistically easier, and provides the longest duration of remission. There is a small risk of therapy-related secondary myelodysplastic syndrome and acute leukemia associated with ASCT.

#### Tandem transplantation

Tandem (double) ASCT refers to a second planned ASCT after recovery from the first transplantation. The role of tandem ASCT in myeloma is limited. A randomized trial conducted in the United States by the Bone Marrow Transplantation Clinical Trials Network (BMT-CTN) has found no benefit with tandem ASCT^41^. However, a survival benefit has been found in a randomized trial conducted by the European Myeloma Network^42,43^. It is likely that the contradictory outcomes in these trials reflect access and availability of new treatment options in the salvage setting. In the US, where multiple options for salvage therapy are available, there seems to be no benefit with tandem ASCT. At present, outside of a clinical trial setting, we consider tandem ASCT only in selected young patients with del 17p.

#### Allogeneic transplantation

Allogeneic transplantation remains investigational in MM. Its use should be restricted primarily to clinical trials, and to young patients (<60 years of age) with high-risk MM that is in first relapse. These patients must be counseled about the high treatment-related mortality rate associated with the procedure and the lack of definitive proof of benefit.

### Consolidation therapy

A randomized trial by the BMT-CTN found no benefit with administering consolidation therapy post ASCT^41^. As a result, we do not recommend additional cycles of VRd chemotherapy or other forms of consolidation following ASCT. Post ASCT, we prefer to move straight to maintenance therapy in the absence of significant residual disease.

### Maintenance therapy

Lenalidomide has been shown to improve PFS and OS following ASCT, and is the recommended form of maintenance for most patients^33,44–48^. Based on the results of the SWOG trial we also recommend lenalidomide maintenance to patients who have not undergone ASCT, but have completed initial therapy with a triplet such as VRd^9^. There is a two- to threefold increase in the risk of second cancers, including therapy-related myelodysplastic syndrome, with lenalidomide and this must be discussed with the patient^44,45^.

In high-risk patients, bortezomib-based maintenance is preferable. In one randomized trial, bortezomib administered every other week as posttransplant maintenance produced better OS than thalidomide maintenance^46^. Bortezomib can be given alone given every other week, or as part of low-intensity VRd, to combine the beneficial effects of bortezomib and lenalidomide^49^. In patients unable to access or tolerate bortezomib, ixazomib is a reasonable alternative that has shown benefit in a placebo controlled randomized trial^50^.

A present data on optimal duration of maintenance are lacking. Long-term indefinite maintenance is associated with cost, toxicity, and inconvenience. Many patients can benefit from a drug-free interval. Currently an ECOG randomized trial is comparing lenalidomide maintenance given until progression versus a limited duration of 2 years. Trials are also examining if the duration of maintenance can be modified based on MRD results. At present we continue maintenance until progression in the absence of toxicity.

## Treatment of relapsed MM

Almost all patients with MM eventually relapse. In fact, MM is a disease characterized by multiple remissions and relapses. With modern therapy, the first relapse of MM occurs after ~3–4 years following initial diagnosis. Each subsequent remission is of shorter duration. Many patients with MM receive five or more lines of therapy in a sequential manner over several years. The remission duration in relapsed MM decreases with each regimen^51^. The choice of treatment at each relapse is affected by many factors. These include the timing of the relapse, response to prior therapy, aggressiveness of the relapse, and performance status. Patients eligible for ASCT should be considered for transplantation if they had elected to delay the procedure, or if they achieved excellent remission duration with the first ASCT, defined as a remission of 36 months or longer with maintenance.

**Fig. 2:**
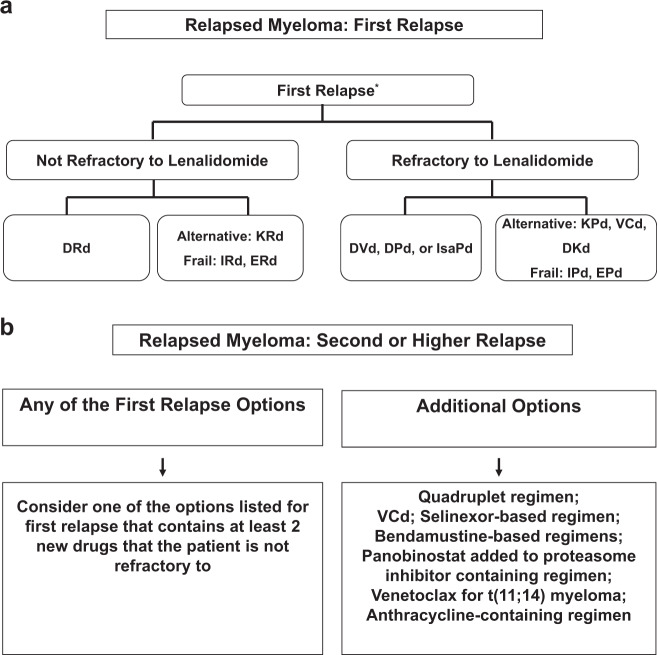
Current Treatment Algorithms for Relapsed Myeloma. Approach to the treatment of relapsed multiple myeloma in first relapse (**a**) and second or higher relapse (**b**). DRd daratumumab, lenalidomide, dexamethasone; KRd carfilozomib, lenalidomide, dexamethasone; IRd ixazomib, lenalidomide, dexamethasone; ERd elotuzumab, lenalidomide, dexamethasone; DVd daratumumab, bortezomib, dexamethasone; DPd daratumumab, pomalidomide, dexamethasone; KPd carfilzomib, pomalidomide, dexamethasone; VCd bortezomib, cyclophosphamide; DKd daratumumab, carfilzomib, dexamethasone; IPd ixazomib, pomalidomide, dexamethasone.

In general, a triplet regimen is preferred. At each relapse, a regimen that contains at least two new drugs that the patient is not refractory to should be considered. Our algorithm for the treatment of relapsed MM is given in Fig. 2. These recommendations are based on the results of several major randomized trials^52–62^. Unfortunately in many of these trials, lenalidomide-containing regimens were tested mainly in patient populations who were not previously exposed to lenalidomide. But in current clinical practice most patients have received lenalidomide as initial therapy, which limits the generalizability of these data to some extent.

### Treatment of first relapse

Patients who are eligible for ASCT should consider ASCT as salvage therapy at first relapse if they have never had a transplant before, or if they have had a prolonged remission with the first ASCT. If relapse occurs more than 6 months after stopping all therapy, the initial treatment regimen that successfully controlled the MM initially can be reinstituted when possible. For all other patients, the strategy for therapy at first relapse is described below. Treatment for relapsed MM is typically continued until disease progression. However, based on tolerability and response, we do consider increasing the interval between cycles, as well as treatment-free intervals. In general, triplet regimens are preferred, but in some elderly frail patients with indolent relapse, doublet regimens such as pomalidomide, dexamethasone can be considered.

At first relapse, for patients who are not refractory to lenalidomide, multiple triplet regimens can be considered, including DRd, KRd, ixazomib, lenalidomide, dexamethasone (IRd), and elotuzumab, lenalidomide, dexamethasone. Each of these regimens has shown superiority over Rd in randomized trials^53,55,56,63^. However, none of them have been compared head-to-head with each other to determine the most effective combination for clinical practice^64^. In the absence of such data, we have to rely on non-randomized comparisons of efficacy and tolerability. Our preferred option is DRd, since it has produced the best reduction in risk of progression compared to Rd, and based on its tolerability. Daratumumab is also available now as a subcutaneous formulation, which reduces infusion-related side effects and reduces the time for administration. KRd is our preferred alternative if daratumumab is not available, or if the patient has been previously treated with daratumumab. For patients who are frail, oral IRd would be a reasonable first choice for relapse.

In patients who are refractory to lenalidomide, options for therapy at first relapse consist of several pomalidomide-based or bortezomib-based combinations. Pomalidomide-based combinations include daratumumab, pomalidomide, dexamethasone, carfilzomib, pomalidomide, dexamethasone (KPd), isatuximab, pomalidomide, dexamethasone, KPd, and elotuzumab, pomalidomide, dexamethasone^62,65–67^. Bortezomib-based combinations include daratumumab, bortezomib, dexamethasone (DVd), VCd, and bortezomib, pomalidomide, dexamethasone^57,68,69^. Daratumumab, carfilzomib, and dexamethasone can also be considered. Unfortunately none of these regimens have been compared head-to-head in randomized trials. Our preferred choice based on non-randomized comparisons of efficacy data is DVd. However, in patients who have been previously treated with daratumumab, KPd would be our preferred option. Any of the other regimens would also be reasonable alternatives depending on availability and comorbidities. As in newly diagnosed MM, VRd and VTd are also active regimens available for use in relapsed disease^70,71^.

Although patients refractory to a drug are likely to be refractory to different drug in the same class, two important exceptions do exist. Pomalidomide has clinical activity in patients who are refractory to lenalidomide^72^, and carfilzomib has activity in patients who are refractory to bortezomib^73^. Carfilzomib is typically administered twice-weekly at a dose of 27 mg/m^2^, but a once-weekly schedule of 56–70 mg/m^2^ may be equally effective and safe, and more convenient^74^. Carfilzomib has a lower risk of neurotoxicity than bortezomib, but ~5% of patients can experience serious cardiac side effects.

### Treatment of second and subsequent relapses

The algorithm for second and subsequent relapses is given in Fig. 2. At each relapse, any of the regimens that were mentioned for use in first relapse can be considered, with the goal of having at least two new drugs that the patient is not refractory two, and preferably from a different drug class. In many instances this may mean the necessity of adding a M antibody to one of the triplets to create a quadruplet regimen. Importantly, alkylator-containing regimens must be considered at this stage. Additional options for second or higher relapses include adding panobinostat to a proteasome-inhibitor containing regimen^58^, or using a selinexor-containing regimen such as selinexor, bortezomib, dexamethasone^75,76^. Bendamustine- or anthracycline-containing regimens are used in refractory settings^77,78^ or the addition of panobinostat to a proteasome-inhibitor containing regimen^58^. For young high-risk patients with a suitable donor, allogeneic transplantation is an option as well^79^.

Venetoclax is not approved for use in MM, but is commercially available, and appears to have single-agent activity in patients with t(11;14) subtype of MM^80^. However, the results of a recent randomized trial found significantly higher mortality with venetoclax in relapsed myeloma despite producing deeper responses and better PFS^81^. Therefore, venetoclax is best considered investigational, and its use should be restricted to patients with t(11;14) who have relapsed disease.

Each remission is likely to be shorter than the previous one. However, with careful analysis of the various options and combinations possible, we can induce remissions multiple times with creative strategies, provided the patient remains in good performance status and is willing and interested in continuing therapy^82^. One strategy we have used in selected patients is one to two cycles of a multi-drug regimen such as VDT-PACE to induce a remission, and then try and maintain it with a more manageable triplet regimen. At each step opportunities for clinical trials may open up and should be considered. Allogeneic transplantation can be considered in selected young patients with relapsed or refractory MM in whom a suitable donor cells are available.

### Investigational treatment approaches

Several promising treatments are under investigation for MM. One of the most exciting options is chimeric antigen receptor T cells targeting B-cell maturation antigen (BCMA) such as bb2121^83^. In studies so far more than 80% of patients appear to respond, with median response duration of ~12 months. Another promising treatment that has been recently approved in the United States is belantamab mafodotin, a humanized anti-BCMA antibody that is conjugated to monomethyl auristatin-F, a microtubule disrupting agent^84^. A third promising new strategy is the use of bispecific T-cell engager, such as AMG 701^85,86^. Cereblon E3 ligase modulators also appear promising^87^.

## Treatment of SMM

SMM is defined by the presence of a serum M protein of ≥3 g/dl (or urine M protein ≥500 mg/24 h) and/or 10–60% BMPCs with no evidence of end-organ damage or other MDE (Table 1)^2,88^. SMM is a clinically defined heterogeneous entity with some patients having biological premalignancy and some with biologic malignancy. Thus some patients behave like M gammopathy of undetermined significance with very low rate of progression (low-risk SMM), while others develop clinical symptoms and end-organ damage within a few years (high-risk SMM)^89^. Although no single pathologic or molecular feature that reliably can be used to distinguish these two groups of patients, we can use a combination of factors to differentiate the two groups (Table 2)^90^. In addition, patients with t(4;14), gain 1q, and del(17p) are at high risk of progression^91,92^.

**Table 2:** Risk stratification of smoldering multiple myeloma (SMM).

**High-risk SMM**
Any 2–3 of the following high-risk factors:
Serum monoclonal protein >2 gm/dL
Serum free light-chain ratio (involved/uninvolved) >20
Bone marrow plasma cells >20%
**Intermediate-risk SMM**
Any 1 high-risk factor
**Low-risk SMM**
No high-risk factor

**Fig. 3:**
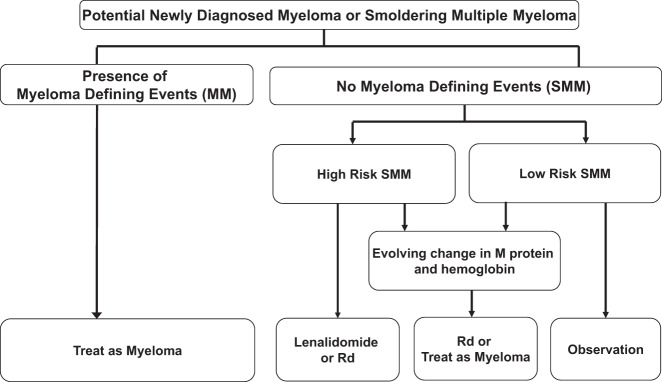
Approach to the management of smoldering multiple myeloma. SMM smoldering multiple myeloma, MM multiple myeloma, Rd lenalidomide plus dexamethasone.

The approach to treatment of SMM is shown on Fig. 3. High-risk patients should be offered therapy with lenalidomide or Rd, or enrollment in a clinical trial. In contrast, patients with low-risk SMM should be observed without therapy every 3–4 months, on an indefinite basis. If during follow up, low-risk SMM patients develop an evolving change in M protein level (defined as 10% increase in M protein within the first 6 months of diagnosis if M-protein 3 g/dl and/or 25% increase in M protein within the first 12 months, with a minimum required increase of 0.5 gm/dl) accompanied by an evolving change in hemoglobin (defined as 0.5 g/dl or greater decrease within 12 months of diagnosis), treatment for MM should be considered according to Fig. 1 or 3. These recommendations are based on data showing that such increase is associated with >90% risk of progression within 2 years^93^

Therapy for high-risk SMM is recommended based on the results of two randomized trials. The first one conducted in Spain found improved PFS and OS with Rd compared with observation in patients with high-risk SMM^94^. In a subsequent ECOG trial, a significant improvement in time to end-organ damage was found with lenalidomide compared with observation^95^. Further studies are comparing a preventive approach (lenalidomide or Rd) as we recommend with treatment with a triplet as in newly diagnosed MM^96^.

Bisphosphonates have been studied in SMM in an attempt to delay bone disease. In a randomized trial, a reduction in skeletal-related events (SRE) has been seen with pamidronate (60–90 mg once a month for 12 months) compared with observation^97^. In a separate randomized trial, zoledronic acid (4 mg once a month for 12 months) reduced SREs compared to versus observation^98^. We feel once-yearly bisphosphonate used for the treatment of osteoporosis is appropriate for most low-risk SMM, but based on data from these two randomized trials, more frequent dosing every 3–4 months can be considered for selected high-risk SMM patients.
